# Regulation of *Bombyx mori*–BmNPV Protein Interactions: Study Strategies and Molecular Mechanisms

**DOI:** 10.3390/v17071017

**Published:** 2025-07-20

**Authors:** Dan Guo, Bowen Liu, Mingxing Cui, Heying Qian, Gang Li

**Affiliations:** 1 Jiangsu Key Laboratory of Sericultural and Animal Biotechnology, School of Biotechnology, Jiangsu University of Science and Technology, Zhenjiang 212100, China; 17385833056@163.com (D.G.); 15580108760@163.com (B.L.); 19513301964@163.com (M.C.); qhysri@just.edu.cn (H.Q.); 2 Key Laboratory of Silkworm and Mulberry Genetic Improvement, Ministry of Agriculture and Rural Affairs, Sericultural Scientific Research Center, Chinese Academy of Agricultural Science, Zhenjiang 212100, China

**Keywords:** *Bombyx mori*, protein–protein interactions, Bombyx mori nucleopolyhedrovirus

## Abstract

As a pivotal model organism in Lepidoptera research, the silkworm (*Bombyx mori*) holds significant importance in life science due to its economic value and biotechnological applications. Advancements in proteomics and bioinformatics have enabled substantial progress in characterizing the *B. mori* proteome. Systematic screening and identification of protein–protein interactions (PPIs) have progressively elucidated the molecular mechanisms governing key biological processes, including viral infection, immune regulation, and growth development. This review comprehensively summarizes traditional PPI detection techniques, such as yeast two-hybrid (Y2H) and immunoprecipitation (IP), alongside emerging methodologies such as mass spectrometry-based interactomics and artificial intelligence (AI)-driven PPI prediction. We critically analyze the strengths, limitations, and technological integration strategies for each approach, highlighting current field challenges. Furthermore, we elaborate on the molecular regulatory networks of Bombyx mori nucleopolyhedrovirus (BmNPV) from multiple perspectives: apoptosis and cell cycle regulation; viral protein invasion and trafficking; non-coding RNA-mediated modulation; metabolic reprogramming; and host immune evasion. These insights reveal the dynamic interplay between viral replication and host defense mechanisms. Collectively, this synthesis aims to provide a robust theoretical foundation and technical guidance for silkworm genetic improvement, infectious disease management, and the advancement of related biotechnological applications.

## 1. Introduction

The silkworm, *Bombyx mori*, is a pivotal economic insect and model organism in Lepidoptera biology. Silkworms lack adaptive immunity and rely solely on innate immunity, rendering them highly susceptible to viral infections. Among these, hemolymphatic infection induced by Bombyx mori nucleopolyhedrovirus (BmNPV) is one of the most prevalent and destructive diseases in sericulture [1]. As a double-stranded DNA virus, BmNPV infection involves intricate host–virus interactions, where protein–protein interactions (PPIs) serve as critical molecular switches orchestrating viral invasion, replication, dissemination, and host immune evasion. Significant advances in *Bombyx mori* proteomics research in recent years have enabled the systematic screening and identification of host–virus PPIs [2,3]. This progress has elucidated the molecular mechanisms underlying key biological processes, including viral infection, immune regulation, and growth [4]. Elucidating these regulatory mechanisms not only deciphers the molecular basis of viral infection but also reveals how viruses hijack host cellular processes for replication and how hosts mount defenses against invasion. These insights provide new targets for developing antiviral strategies in silkworms, thereby facilitating genetic improvement and disease control in sericulture. This review systematically summarizes research progress on silkworm–virus interaction proteins, focusing on experimental and bioinformatics methodologies, underlying molecular mechanisms and translational applications. It aims to provide a scientific foundation for developing novel antiviral control strategies.

## 2. Techniques for Protein Interaction Research: From Classics to Frontiers

### 2.1. Traditional Protein Interaction Research Techniques

Protein–protein interaction (PPI) refers to the non-covalent binding of two or more protein molecules within a cell to form complexes that participate in vital biological processes, such as development, signal transduction, and metabolism. PPIs encompass not only direct protein–protein binding but also interactions between proteins and nucleic acids (DNA/RNA), with the latter playing a critical role in regulating gene transcription and expression. Traditional techniques for studying protein interactions, including yeast two-hybrid (Y2H), GST pull-down, co-immunoprecipitation (Co-IP), and bimolecular fluorescence complementation (BiFC), have been fundamental in uncovering protein interaction networks in *Bombyx mori*. These methods have not only elucidated key interaction mechanisms through the direct capture of protein complexes or functional validation but also established a foundational framework for the development of high-throughput screening technologies.

The Y2H system is based on the principle of using transcription factor recombination to activate reporter gene expression and is widely employed in the study of virus infection and host interactions [5,6]. Typical applications in the study of genomic interaction proteins in the silkworm include the screening of midgut proteins interacting with Bombyx mori densonucleosis virus–Zhenjiang isolate (BmDNV-Z) [7]; resolving the mechanism of interaction between BmNPV oral infectious factor P74 (P74) and after-occlusion-derived virus (ODV)-bound host proteins [8]; and unraveling the interactions between viral vesicle membrane protein GP64 and E3 ubiquitin–protein ligase SINA-like 10 (SINAL10) [9].

Co-immunoprecipitation (Co-IP) is one of the most widely used methods for identifying interactions between novel proteins or complexes formed by known proteins [10]. Enriching protein complexes with specific antibodies has validated its utility in verifying the interaction between the microsporidian spore wall protein 25 (SWP25) of Microtus sphaericus and the turtle-like protein of *B. mori* (BmTLP) [11], as well as in revealing the interaction between transport protein SEC61 (ID:Q19AA9) and phosphate carrier protein PIC (ID:Q1HPL2) in promoting viral proliferation [12]. Using Co-IP, Zhang et al. validated 10 protein interactions initially identified by the Y2H system [13], confirming it as an essential method for verifying protein interactions.

The GST pull-down technique is based on the high-affinity capture of protein complexes by glutathione-S-transferase (GST) tags with glutathione (GSH). It is commonly used to detect protein interactions in vitro [14]. For example, GST pull-down combined with mass spectrometry techniques was used to identify candidate proteins interacting with the protein tyrosine phosphatase of the BmNPV-PTP brain [15].

Bimolecular fluorescence complementation (BiFC) visualizes live-cell interactions through the recombination of fluorescent protein fragments and has been used to reveal the functional association of cell cycle-dependent kinase 11 (CDK11) with the RNA splicing factors RNPS1 and 9G8 in *Bombyx mori* [16,17], as well as the molecular pattern of lincRNA_XR209691.3 and the silkworm heat shock protein 70 (HSP70) in synergistically regulating the molecular pattern of viral replication [18]. Together, these classical approaches have constructed a scientific model for studying protein interactions in *Bombyx mori*.

### 2.2. Emerging Protein Interaction Research Techniques

Although classical protein interaction research techniques (e.g., yeast two-hybrid and immunoprecipitation) have provided a foundational framework for studying protein interactions in *Bombyx mori*, they inherently suffer from limitations in throughput, spatiotemporal resolution, and dynamic interaction capture. In recent years, rapid advancements in technologies such as mass spectrometry (MS), surface plasmon resonance (SPR), and artificial intelligence (AI) have propelled protein interaction research into a new era of high-throughput, high precision and dynamic resolution.

Mass spectrometry (MS) leverages molecular ionization and mass-to-charge (*m*/*z*) separation, enabling protein identification and interaction analysis through signal intensity-based molecular mass determination and structural characterization [19]. Liquid chromatography–tandem mass spectrometry (LC-MS/MS) integrates high-specificity identification with precise quantification. For example, Guo et al. used LC-ESI-MS/MS to identify the co-localization of silkworm HSP70-4 with BmNPV polycomb proteins, revealing the critical regulatory role of the HSP70 family in viral replication cycles [20].

SPR technology enables label-free, real-time dynamics for molecular interactions by detecting angular shifts induced by binding events [21]. Its advantages—including label-free detection, real-time analysis, high sensitivity, and operational simplicity—make it ideal for profiling dynamic interactions [22]. Utilizing SPR, Jeremy et al. characterized the specific binding of *Bacillus thuringiensis* Cry1Aa toxin to silkworm midgut receptors, *Bombyx mori* aminopeptidase N (BmAPN), and BmCadherin-like, providing a theoretical foundation for developing targeted pest control strategies [23].

AI-driven protein interaction prediction, powered by deep learning models (e.g., AlphaFold-Multimer and D-SCRIPT), predicts protein interaction interfaces and complex structures with high throughput, low costs, and scalability for large-scale network inference. Ramasamy Sumathy constructed a silkworm PPI network using the Interlog method, validating predictions through the integrative analysis of iPfam interaction domains and gene expression data [24].

Collectively, these emerging technologies address the limitations of traditional methods, providing novel approaches to unraveling the complexity of silkworm protein interaction networks and their dynamics in antiviral defense and developmental regulation.

### 2.3. Technology Integration Strategies and Challenges

#### 2.3.1. Current Challenges

Advances in protein–protein interaction (PPI) research rely on the complementary strengths of both traditional and emerging methodologies (Table 1). Traditional approaches, while operationally simple and cost-effective, are limited by several factors, including reduced sensitivity (e.g., Y2H is restricted to nuclear interaction detection [25]), high false-positive rates (Co-IP is constrained by antibody specificity [26]; GST pull-down is susceptible to endogenous protein interference [27]), low throughput (GST pull-down struggles with high-throughput screening [27]), and the inadequate capture of dynamic interactions (Y2H fails to detect weak/transient binding events [25]). By contrast, emerging technologies enhance resolution and dynamic monitoring capabilities. For example, cross-linking mass spectrometry (XL-MS) resolves the interaction interfaces and spatial conformations of protein complexes, while SPR enables the label-free, real-time tracking of interaction kinetics [28,29]. However, these methods face challenges such as technical complexity, reliance on specialized equipment, and data analysis handling. Combining traditional and emerging techniques significantly deepens research depth and reliability, providing robust tools for comprehensive protein interaction network analysis [30]. Researchers must select appropriate methods or employ multi-technique integration based on specific scientific questions and experimental contexts to obtain holistic interaction data. To address these challenges, optimizing bait and prey protein expression/construction is pivotal for reducing false positives. Inconsistencies between Y2H-screened candidate interactors and Co-IP validation hinder accuracy. Additionally, the high cost and technical complexity of emerging technologies (e.g., cross-linking mass spectrometry and live-cell imaging) limit accessibility, prompting efforts to reduce costs and simplify workflows.

#### 2.3.2. Technology Integration Strategies

The accuracy and efficiency of protein–protein interaction (PPI) research depend on the systematic integration of traditional techniques, emerging methods, and process optimization. Current methodologies still face challenges, including high false-positive rates, insufficient capture of dynamic interactions, and difficulties in data integration. To address these issues, we propose the following optimization strategies.

To address the high false-positive rate of Y2H screening, we established a three-stage verification system: (1) primary screening employing Y2H (e.g., a gateway system) for large-scale candidate identification, augmented by multi-AI structural compatibility scoring to reduce false positives [24]; (2) secondary screening using GST pull-down for in vitro validation to exclude yeast-specific artifacts, coupled with bimolecular fluorescence complementation (BiFC) for subcellular colocalization analysis [14,16]; and (3) confirmation via endogenous Co-IP/LC-MS/MS to verify physiological interactions, with SPR quantifying binding kinetics (e.g., KD) [10]. This system is optimally suited for verifying stable, high-abundance proteins forming persistent complexes in specific cellular compartments, whereas PTM-dependent or transient interactions (e.g., signaling proteins) require cautious interpretation and functional validation to confirm physiological relevance.

To overcome limitations in capturing transient interactions, we integrate time-resolved sampling with in situ analysis: cross-linking mass spectrometry (XL-MS) applied to samples across infection timepoints captures ephemeral complexes, with comparative network analysis elucidating the temporal mechanisms of viral invasion [28]. Concurrently, live-cell imaging (confocal microscopy with fluorescent tagging) coupled with BiFC tracks the real-time colocalization dynamics of viral (e.g., GP64) and host proteins (e.g., BmREEPa) at the plasma membrane, resolving spatial interaction specificity [18]. This integrated strategy can successfully dissect BmNPV nucleocapsid nuclear entry: for example, XL-MS was used to identify a transient Hsp90–tubulin complex at 2 hpi, while live imaging confirmed its role in microtubule-mediated nucleocapsid transport.

## 3. Identification and Functional Analysis of *Bombyx mori* Proteins Interacting with BmNPV and Their Roles in Viral Proliferation

The molecular regulatory mechanisms governing *Bombyx mori*–BmNPV interactions operate across multiple dimensions, each orchestrating viral proliferation through distinct protein networks. Elucidating these mechanisms is essential for understanding insect antiviral immunity and viral pathogenesis and developing targeted control strategies. These interactions exhibit dynamic multidimensional complexity, spanning intracellular processes, gene expression regulation, and systemic immune responses—all interconnected through reciprocal regulation. Key interaction mechanisms involve (1) apoptosis and cell cycle control, (2) viral protein invasion/trafficking, (3) non-coding RNA regulation, (4) metabolic reprogramming, and (5) immune signaling pathways.

### 3.1. Apoptosis and Cycle Regulation

#### 3.1.1. Apoptosis Regulation

Apoptosis is a critical component of the insect immune response, serving as an effective defense against viral replication and proliferation. The conserved presence of the *IAP* gene in both host and virus genomes suggests its pivotal role in virus–host interactions, offering a key entry point for dissecting molecular mechanisms. First, we turn to pro-apoptotic defense mechanisms: The *Bombyx mori apoptosis regulator* (*BmP53*) directly activates apoptosis, while its interaction with the inhibitor of growth protein 5 (ING5) accelerates apoptosis by decreasing the mitochondrial membrane potential, thereby promoting viral particle release and clearance [39,40]. Conversely, acetylated ING5 reduces P53 stability and reverses its pro-apoptotic function [41], highlighting the fine regulation of epitope modifications in balancing apoptosis and viral proliferation. Second, apoptosis inhibition promotes viral proliferation: viruses sustain infected cell viability to facilitate replication through the synergistic action of BmIAP and serine/threonine phosphatase 5 (BmPP5), which together block caspase cascade [4].

#### 3.1.2. Cell Cycle Regulation

**Figure 1:**
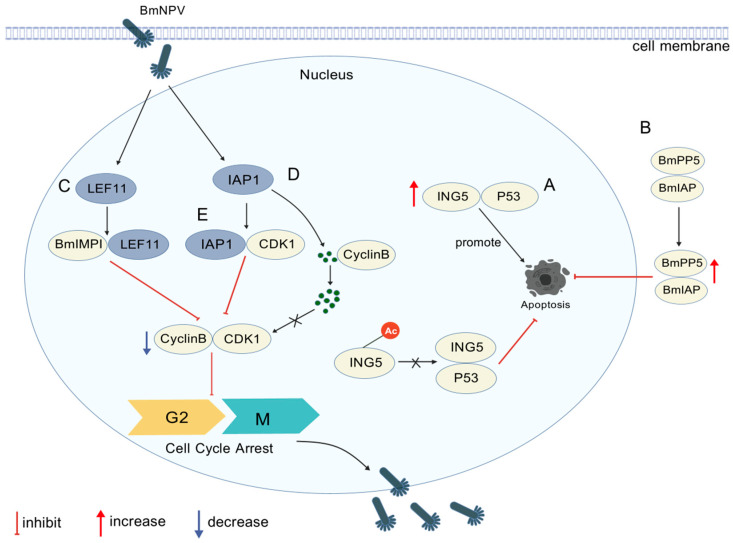
Molecular mechanisms of protein interactions in apoptosis and cycle regulation of BmNPV proliferation (created with biogdp.com). (**A**) The interaction between BmIAP and BmPP5 promotes the expression of both proteins and inhibits host cell apoptosis. (**B**) BmING5 interacts with P53 to inhibit cell proliferation and promote apoptosis; ING5 acetylation weakens this interaction, reducing P53 stability to enhance proliferation and suppress apoptosis in BmN cells. (**C**) *LEF-11* interacts with BmIMPI and leads to cell cycle arrest in the G2/M phase by inhibiting CDK1/cyclin B complex activity. (**D**) BmNPV IAP1 promotes the nuclear accumulation of BmCyclin B, disrupting functional cyclin B-CDK1 complex formation by blocking cytoplasmic assembly or inducing the premature nuclear translocation of inactive complexes, thereby causing G2/M phase arrest via impaired mitotic entry. (**E**) BmNPV IAP1 specifically targets BmCDK1 to suppress its expression post-infection, impairing the nuclear accumulation of the BmCyclin B–CDK1 complex and triggering G2/M phase arrest through mitotic transition failure.

In *Bombyx mori*, cell cycle regulation is intricately linked to BmNPV proliferation. A key mechanism involves G2/M phase arrest, where BmNPV manipulates the cell cycle to facilitate viral replication. *Baculovirus late-expressed factor 11* (*LEF-11*) interacts with the developmental immune-associated protein BmIMPI, inducing G2/M arrest by modulating host cell cycle proteins, such as by inhibiting cyclin-dependent kinase 1 (CDK1)/cyclin B complex activity [42]. Concurrently, the viral protein IAP1 interacts with BmCyclin B, a critical regulator of the G2/M transition, causing the aberrant nuclear accumulation of cyclin B and specifically blocking the cell cycle at the G2/M phase to create a temporal window for viral replication [43]. Additionally, IAP1 associates with the cell cycle kinase BmCDK1, reducing BmCDK1 levels to enforce BmNPV-induced G2/M arrest and support viral proliferation [44]. The molecular mechanisms of protein interactions involved in apoptosis and cycle regulation during BmNPV proliferation are illustrated in Figure 1 and Table 2. In summary, apoptosis and cell cycle regulation are central to the *Bombyx mori*–BmNPV interaction. Through multi-protein interactions, the virus and host engage in a dynamic interplay that activates or inhibits apoptosis and arrests or drives the cell cycle. These mechanisms provide a theoretical foundation for developing antiviral interventions targeting host factors such as CDK inhibitors and apoptosis pathway activators.

### 3.2. Regulation of Viral Protein Invasion and Transport

#### 3.2.1. Viral Invasion and Membrane Fusion: Vesicle Protein-Mediated Adsorption and Membrane Fusion

BmNPV virus particles bind to specific receptors on the host cell surface via vesicular membrane proteins. For instance, the vesicle membrane protein GP64 initiates viral adsorption by binding to host membrane cholesterol through its cholesterol recognition motif (CRAC) [45]. During the membrane fusion stage, GP64 undergoes K63-linked ubiquitination mediated by SINAL10 and interacts with heat shock protein 75 kDa (TRAP1), stabilizing its conformation to promote endosomal membrane fusion [9,46]. The *Bombyx mori* receptor expression-enhancing protein (BmREEPa) directly binds to BmNPV envelope protein GP64 through its TUBBY structural domain, enhancing the efficiency of GP64-mediated virus–host membrane fusion [47]. The host membrane remodeling complex BmREEPa/BmPtchd anchors the transmembrane region (TM) and extracellular domain (ECD) of GP64 via their TUBBY structural domains, forming a ternary complex that collectively optimizes the efficiency of viral envelope fusion and endocytosis [48]. *Bombyx mori* Niemann–Pick type C (BmNPC1) is a key receptor that mediates the endocytosis of viral GP64 [49]. During endocytosis, the NPC1–NPC2 receptor complex acts in concert with membrane fusion to mediate viral internalization, and mutations in its interaction sites significantly inhibit viral proliferation [50,51]. The host transporter protein SEC61 promotes viral replication by mediating viral protein translocation, whereas the retinoic acid-binding protein FABP1 (ID:Q2QEH2) inhibits GP64 membrane fusion by antagonizing E3 ubiquitinase activity [12]. Furthermore, the nucleocapsid protein VP39 interacts with F-actin, potentially interfering with viral transport, although its direct antiviral mechanism requires further verification [52].

#### 3.2.2. Viral Transport and Nucleation: Microtubule Network-Dependent Intranuclear Transport

**Figure 2:**
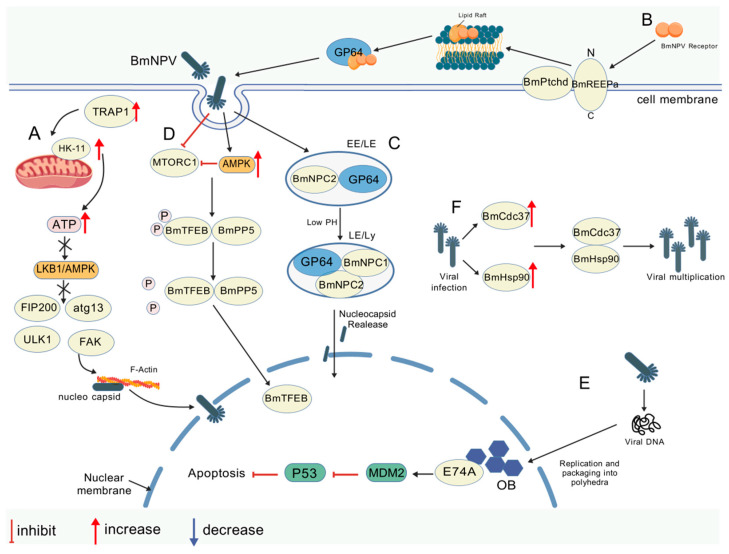
Molecular mechanisms of protein interactions in viral invasion and transport-regulating BmNPV proliferation (created with biogdp.com). (**A**) TRAP1 upregulates HK-II to boost ATP biosynthesis, suppresse LKB1/AMPK, disrupt ULK1-FIP200-FAK complexes, release monomeric FAK for actin polymerization, and facilitate BmNPV nucleocapsid nuclear entry. (**B**) BmREEPa, located on the cell membrane with its N-terminal extracellular and C-terminal cytoplasmic, recruits BmNPV receptors into plasma membrane rafts to form complexes that interact with GP64, facilitating virus particle entry via receptor-mediated endocytosis during BmNPV infection. (**C**) The virus binds to NPC2 and cell attachment factors to adhere to the cell membrane and translocates to the LE/Ly compartment, where low pH induces structural changes in GP64 and NPC1 to expose binding sites, enabling BmNPV GP64 to interact with NPC1/NPC2 for viral envelope–endosomal membrane fusion and nucleocapsid release into the cytoplasm. (**D**) BmNPV infection promotes its proliferation in silkworms by preventing MTORC1 from upregulating competing protein kinase 1, which activates AMPK signaling to induce dephosphorylation and the cytoplasmic–nuclear translocation of BmTFEB. (**E**) During the late-infection viral assembly of ODV in the nucleus, Polycomb interacts with BmE74A to potentially promote BmNPV proliferation by regulating downstream BmMdm2 and Bmp53 expression, while BmCdc37 directly interacts with BmHsp90 to form a chaperone complex that enhances viral proliferation. (**F**) BmCdc37 and BmHsp90 both promote BmNPV reproduction, with their co-expression enhancing virus proliferation more effectively than individual overexpression.

Transporting the BmNPV viral nucleocapsid to the nucleus is a crucial step for genome replication and transcription initiation and relies on the cellular microtubule network. BmHsp90 interacts with microtubule-binding structural domain protein (BmTbce) and Golgi subfamily A member 5 (BmGolga5), facilitating the retrograde transport of the virus into the nucleus [53,54]. Protein kinase 1 (PK1) activates the AMPK signaling pathway in silkworms, which mediates the dephosphorylation of BmPP5, enabling its binding to *transcription factor EB* (*BmTFEB*). This interaction drives BmTFEB nuclear translocation and activates the expression of viral proliferation-associated genes, such as nucleotide synthases [55]. The *transcription factor BmE74A* (*BmE74A*) is a member of the erythroblast transformation-specific (ETS) family of transcription factors in *Bombyx mori* and directly binds to viral proteins, promoting viral proliferation [56]. *Bombyx mori* cell division cycle protein 37 (BmCdc37), functioning as a molecular chaperone, interacts with BmHsp90 to enhance its activity, thereby supporting the proper folding of viral proteins [57]. The very early protein PE38 interacts with the *Bombyx mori eukaryotic translation initiation factor* (*BmeIF4E*) and the splicing kinase *Bombyx mori* SRSF protein kinase 1-like (BmSRPK), promoting early viral gene expression [58,59]. The inclusion of body-derived virus particle (ODV) capsid protein P74 mediates invasion by binding to midgut JAB-MPN structural domain proteins, which are essential for viral transport and diffusion within host cells and may participate in interactions between the viral nucleocapsid and cytoskeletal or transport proteins [8]. By contrast, at the host defense level, *Bombyx mori* thymopeptide (BmTHY) binds to actin, inhibiting BmNPV proliferation, likely by interfering with viral transport, and subsequently suppressing nucleocapsid migration and replication [60]. The molecular mechanisms of protein interactions involved in viral invasion and transport-regulating during BmNPV proliferation are illustrated in Figure 2 and Table 3.

### 3.3. Non-Coding RNA Regulation

#### 3.3.1. Mechanisms of Non-Coding RNA-Mediated Viral Proliferation Promotion

RNA sedimentation, mass spectrometry, truncation, and RNA immunoprecipitation (RIP) assays have revealed that lincRNA_XR209691.3 binds to the actin-binding domain of silkworm heat shock protein 70 (BmHSP70), stabilizing this host factor. This interaction improves viral protein folding efficiency and promotes BmNPV replication and translation [18]. Transcriptome sequencing and ribosome profiling (Ribo-seq) have revealed significant lnc557 upregulation during BmNPV infection. Subcellular fractionation assays have indicated its cytoplasmic enrichment. RNA pull-down, coupled with mass spectrometry, truncation, and RIP assays, has demonstrated that lnc557 binds the RRM5 domain of BmELAVL1, enhancing its stability. Overexpression of BmELAVL1 promotes BmNPV proliferation, while its knockdown suppresses viral replication, indicating that lnc557 facilitates viral proliferation by stabilizing BmELAVL1 [61]. By contrast, BmNPV infection downregulates lnc_209997 expression. Dual-luciferase reporter assays confirm that lnc_209997 directly interacts with miR-275-5p. Downregulating lnc_209997 releases miR-275-5p, which targets host/viral transcripts to modulate signaling pathways and promote viral proliferation [62].

#### 3.3.2. Non-Coding RNA-Mediated Host Antiviral Defense Mechanisms

MiR-3351 was identified via transcriptome sequencing combined with miRNA-mRNA association analysis. Dual-luciferase reporter assays validated its direct binding to the 3′UTR of glutathione S-transferase epsilon 6 (BmGSTe6) [63]. RNA fluorescence in situ hybridization (FISH) revealed their co-localization in the cytoplasm, and their interaction was confirmed by RNA immunoprecipitation (RIP). MiR-3351 modulates glutathione content by downregulating BmGSTe6, thereby inhibiting BmNPV proliferation in *Bombyx mori*. MiR-6498-5p, identified as being differentially expressed via transcriptome sequencing of BmNPV-infected midguts, is downregulated upon BmNPV infection (RT-qPCR). Its target BmPLPP2 (pyridoxal phosphate phosphatase 2) is conversely upregulated, showing a negative correlation. Dual-luciferase reporter assays and in vivo RNA immunoprecipitation (RIP) have confirmed the direct binding of bmo-miR-6498-5p to the BmPLPP2 coding sequence (CDS), thereby negatively regulating BmPLPP2 and inhibiting PLP dephosphorylation [64].

#### 3.3.3. Strategies for Identifying BmNPV-Derived ncRNAs and Their Host Targets

BmNPV-derived non-coding RNAs (ncRNAs) are primarily identified through multi-layered approaches combining deep sequencing and experimental validation. Deep sequencing serves as the core foundation: total RNA sequencing is performed on BmN cells or silkworm tissues at different infection stages, with strand-specific sequencing to distinguish sense and antisense transcripts, facilitating the identification of viral antisense RNAs or promoter-associated ncRNAs [65]; small RNAs of 18–30 nt are enriched to identify viral miRNAs, with analyses requiring alignment to the BmNPV genome to differentiate viral from host miRNAs [66]. Additionally, virus-specific enrichment strategies, such as capture sequencing using BmNPV genome probes, enable the efficient enrichment of viral ncRNAs during early infection or at low titers, enhancing detection sensitivity.

**Figure 3:**
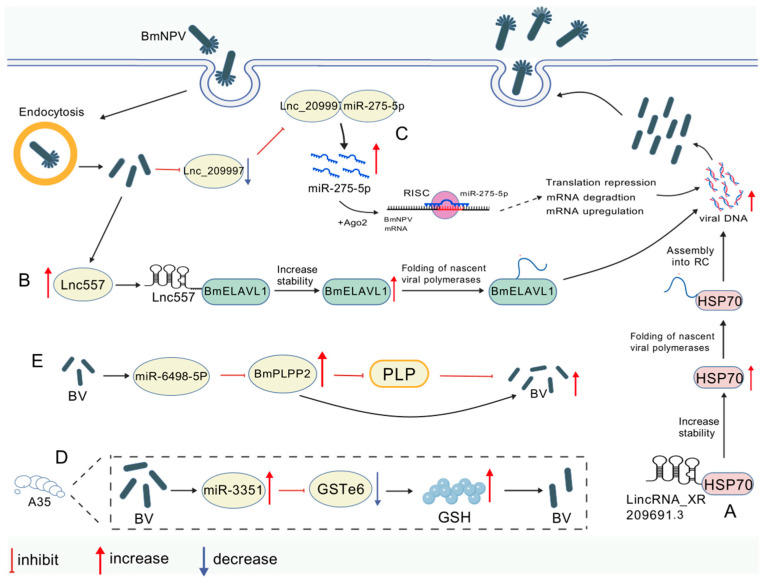
Molecular mechanism of protein interactions involved in non-coding RNA regulation of BmNPV proliferation (created with biogdp.com). (**A**) During BmNPV infection, upregulated lincRNA_XR209691.3 stabilizes BmHSP70 by binding to its actin-binding domain, promoting BmHSP70 accumulation to enhance viral protein folding/maturation and facilitate viral proliferation. (**B**) Lnc557 binds to the RRM-5 structural domain of BmELAVL1 and promotes BmELAVL1 expression by enhancing its stability, thereby promoting BmNPV proliferation. (**C**) BmNPV infection suppresses Lnc_209997 expression, attenuating its interaction with miR-275-5p and liberating miR-275-5p to dysregulate PI3K-AKT/MAPK signaling pathways. This miR-mediated pathway hijacking enhances viral proliferation by rewiring host–viral gene networks. (**D**) In BmNPV-resistant strain A35, miR-3351 is upregulated and GSTe6 is downregulated after BmNPV infection, and more GSH leads to reduced BmNPV. (**E**) Silkworm miR-6498-5p targets BmPLPP2 phosphatase mRNA to suppress PLP dephosphorylation, depleting PLP and disrupting viral coenzyme metabolism, thereby inhibiting BmNPV infection.

For the experimental validation of host targets, Northern blotting verifies the size and expression kinetics of candidate ncRNAs, while RT-qPCR enables high-throughput validation of their expression levels, tissue specificity, and dynamic changes during viral infection. Bioinformatics analysis is also critical: sequencing reads are aligned to both host and viral genomes; novel transcripts are assembled and identified; differential expression analyses are conducted; and ncRNA structural/functional domains are predicted using databases such as Rfam, which help distinguish virus-derived from host-derived reads and enrich them for significant biological functions and signaling pathways. RNA immunoprecipitation sequencing (RIP-seq) can analyze host target mRNAs; for specific lncRNAs, biotinylated probes can enrich associated DNA, RNA, or proteins, enabling the identification of genomic binding sites or the RNA targets of lncRNAs. The validation of lncRNA-miRNA interactions employs three methods: first, the luciferase reporter system, in which bioinformatics analysis identifies candidate lncRNA binding regions, and wild-type and binding site-mutated 3′-UTR sequences are cloned into commercial luciferase reporter vectors, with lncRNA-induced changes in luminescence intensity used to verify binding sites; second, the biotin–avidin pull-down system, in which qPCR is performed on lncRNAs enriched via pull-down to quantify and verify the specific binding between miRNAs and lncRNAs; and third, q-PCR techniques, such as the stem-loop and polyadenylation methods, which detect miRNAs and their target mRNAs. The molecular mechanisms of protein interactions involved in non-coding RNA regulation during BmNPV proliferation are illustrated in Figure 3 and Table 4.

### 3.4. Metabolic Regulation

#### 3.4.1. Mitochondrial Metabolic Antiviral Mechanisms

Upon pathogen infection, the energy and material metabolic pathways in silkworms are reprogrammed to meet the bioenergetic and biosynthetic demands of the immune response. Mitochondria, as the core hub of metabolism and apoptosis regulation, orchestrate a dynamic interplay between host defense and viral counter-strategies. The *Bombyx mori* ADP/ATP translocase (BmANT) downregulates the expression of the heat shock protein (BmHSP60) [54], inhibiting viral replication via reciprocal interaction, which may suppress host cell apoptosis during viral invasion by inhibiting apoptotic signaling. The formation of the BmANT-VDAC inner membrane complex disrupts mitochondrial respiratory function by regulating ADP/ATP transport, whereby the aberrant accumulation of BmANT triggers ATP/ADP transport dysfunction to inhibit BmNPV proliferation and replication [67]. Concurrently, the release of Bmcytc activates mitochondrial apoptotic cascades (e.g., Bmapaf and Bmcaspase-Nc), eliminating infected cells via programmed death to restrict viral dissemination [68].

#### 3.4.2. Mechanisms of Mitochondrial Virus Hijacking

**Figure 4:**
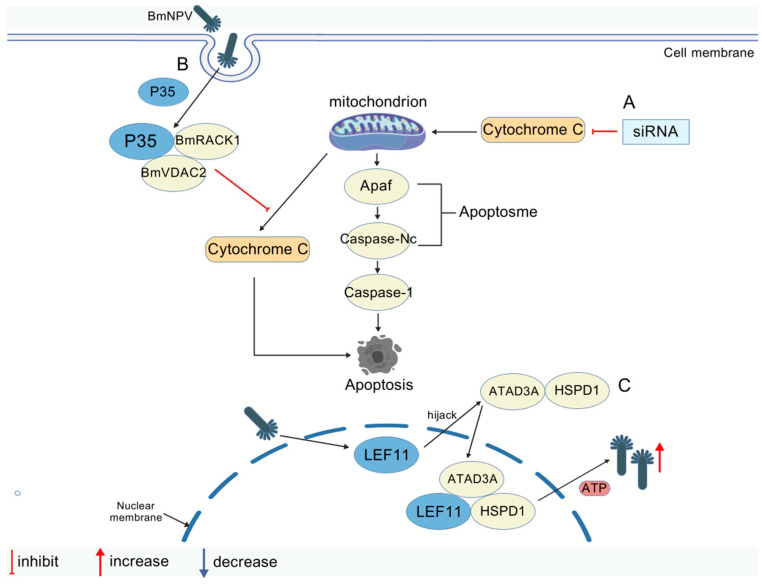
Molecular mechanisms of protein interactions in mitochondrial metabolism regulating BmNPV proliferation (created with biogdp.com). (**A**) Bmcytc RNAi activates the mitochondrial apoptotic pathway, where Bmcytc and downstream key genes promote apoptosis in infected cells. (**B**) BmNPV p35 interacts with the VDAC2-RACK1 complex to inhibit cytochrome C release, thereby regulating apoptosis. (**C**) Following viral invasion, the *LEF-11* protein hijacks host ATPase family members ATAD3A and HSPD1 to form a ternary complex, providing ATP for viral replication.

In apoptotic pathway inhibition, the BmNPV-encoded p35 protein targets the host BmVDAC2-BmRACK1 complex (mitochondrial protein-scaffolding complex), blocking cytochrome C release and suppressing mitochondria-dependent apoptosis to sustain infected cell viability for viral proliferation [69]. In energy metabolism hijacking, *LEF-11* activates host ATPase and enhances mitochondrial OXPHOS to facilitate viral DNA replication via interactions with the mitochondrial inner membrane ATPase ATAD3A and the heat shock protein HSPD1 (Hsp60) [70]. Additionally, the phosphate carrier protein PIC provides mitochondrial energy support for the virus, leading to further promotion [13]. The bidirectional regulation of mitochondrial function by the host and virus reveals the dual role of metabolic regulation in the interaction between the silkworm and BmNPV. Studies have provided a theoretical framework for developing antiviral strategies targeting mitochondrial metabolic nodes, such as ATAD3A inhibitors. The molecular mechanisms of protein interactions involved in mitochondrial metabolism regulating during BmNPV proliferation are illustrated in Figure 4 and Table 5.

#### 3.4.3. Immunomodulation

Small ubiquitin-associated modifier (SUMO) is a ubiquitin-like protein that is modified by SUMOylation through covalent binding to the lysine residues of target proteins. This post-translational modification process plays an important role in key cellular functions, such as DNA repair, intracellular trafficking, signal transduction, and stress response. *Bombyx mori* builds multilayered antiviral defenses through SUMOylation modification and innate immune pathways. The translation-controlled tumor protein BmTCTP not only enhances its own stability after SUMOylation through the SUMOylation-conjugating enzyme BmUBC9/BmSMT3 but also interacts with the interleukin enhancer-binding factor BmILF to synergistically activate downstream immune signals and significantly inhibit viral replication [71].

**Figure 5:**
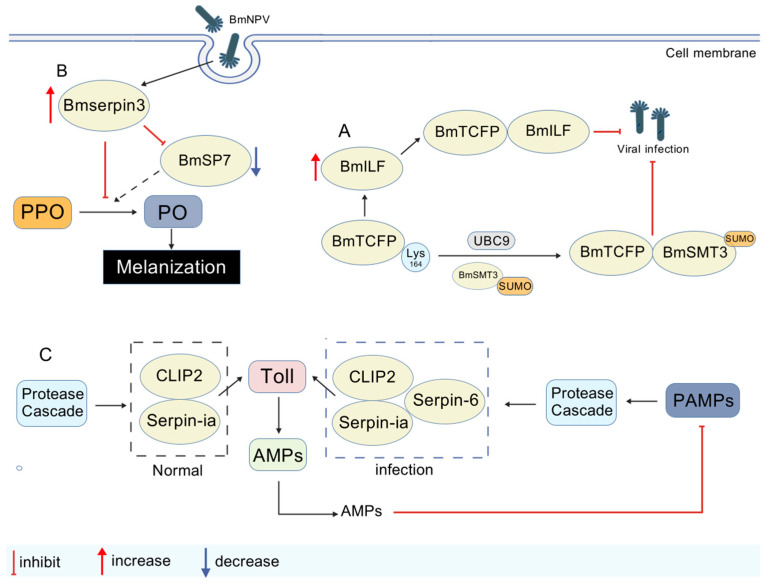
Molecular mechanisms of protein interactions in immune system regulation of BmNPV proliferation (created with biogdp.com). (**A**) BmTCTP undergoes SUMO methylation at lysine 164 via BmUBC9/BmSMT3, forming a stabilized BmTCTP-BmSMT3 fusion protein. This complex physically interacts with BmILF, upregulating BmILF expression during viral infection to enhance antiviral activity. (**B**) BmSerpin3 achieves PO inhibition by suppressing PPO activation and interacting with BmSP7 to regulate the activation cascade. (**C**) Increased CLIP2 activation in the hemolymph induces serpin-6 expression, which collaborates with serpin-1a to dampen excessive CLIP2 activity, preventing over-activation of the Toll pathway and sustaining immune balance.

In the innate immune system of *Bombyx mori*, the phenoloxidase (PPO) activation pathway, initiated by a serine protease cascade, triggers melanization, which serves to eliminate invading pathogens [1]. The serine protease inhibitor BmSerpin3 exhibits dual regulatory roles: (1) it interacts with storage proteins (e.g., sex-specific storage protein 2) to modulate immune responses [72]; and (2) it irreversibly inhibits serine protease 7 (SP7) via its Reactive Center Loop (RCL), thereby precisely regulating the PPO activation cascade and balancing melanization intensity with the risk of potential tissue damage [73]. Another inhibitor, BmSerpin2, may exert anti-BmNPV effects by regulating PPO activity and suppressing melanization [74]. In addition, silkworm lipases and serine proteases have been shown to possess antiviral activity against BmNPV [75]. The Toll pathway has been shown to possess antiviral activity against BmNPV [76]. The serine protease CLIP2 activates the Toll pathway by cleaving the extracellular ligand proSpätzle1, thereby upregulating AMP expression and enhancing the antimicrobial activity of the hemolymph. Constitutive serpin-1a and inducible serpin-6 expression synergistically inhibit CLIP2 via covalent complex formation, maintaining immune homeostasis [77]. Collectively, antiviral immunity in *B. mori* involves dynamic regulation across multiple tiers and pathways, with its core mechanism rooted in the host’s capacity to establish a dual barrier of defense and immune balance through the intricate coordination of post-translational modifications and innate immune pathways. The molecular mechanisms of protein interactions involved in immune system regulation during BmNPV proliferation are illustrated in Figure 5 and Table 6.

## 4. Discussion

### 4.1. Comparative Insights with Closely Related Baculoviruses

Baculoviruses, such as Autographa californica multicapsid nucleopolyhedrovirus (AcMNPV), share conserved core replication machinery with BmNPV but exhibit distinct host specificities and interaction strategies, providing valuable comparative insights into virus–host protein interactions.

In terms of viral invasion and membrane fusion, both BmNPV and AcMNPV rely on the envelope glycoprotein GP64 for host cell entry [78], but their receptor interactions differ. BmNPV GP64 binds to host factors such as the BmREEPa/BmPtchd complex and BmNPC1/NPC2 for endocytosis and fusion [47,48]. By contrast, AcMNPV GP64 mediates baculovirus entry into mammalian cells by binding to heparan sulfate proteoglycans on the cell surface, initiating dynein- and calreticulin-dependent endocytosis [79], which is one of the reasons why AcMNPV can infect multiple lepidopteran species. This divergence reflects the adaptation of BmNPV to the unique membrane components of *Bombyx mori*, highlighting host-specific co-evolution in viral entry mechanisms. Furthermore, studies indicate that, besides BmNPV, the Helicoverpa armigera nucleopolyhedrovirus (HaNPV) Ha-VP39 can bind actin directly and polymerize to drive retrograde nucleocapsid transport without cofactors [80], reconfiguring the cytoskeleton for motility. However, it lacks intrinsic nuclear localization signals and depends on host factors for nuclear entry [81,82]. The precise molecular mechanism of the interaction between BmNPV VP39 and host F-actin remains unclear and warrants further investigation.

The broad host range of AcMNPV establishes it as a versatile vector for protein expression, whereas the specificity of BmNPV enables targeted silkworm disease control. Comparative studies can identify both pan-baculoviral targets (e.g., HSP90) for broad-spectrum antiviral strategies and species-specific factors (e.g., BmNPC1) for precise interventions.

### 4.2. Unresolved Challenges and Future Prospects

The interplay between BmNPV and its host, the silkworm, involves complex and dynamically regulated protein interactions, although key questions remain unresolved. For instance, host-encoded E3 ubiquitin ligase SINAL10 promotes GP64-mediated viral membrane fusion, while the host protein FABP1 counteracts this by inhibiting E3 ubiquitinase activity, thereby blocking GP64 membrane fusion. A critical next step is to elucidate whether SINAL10 promotes viral proliferation independently or synergistically with GP64 at the molecular level [9].

Secondly, the viral protein IAP1 is hypothesized to exert dual functions—including cell cycle arrest in the nucleus [44] and inhibiting apoptosis in the cytoplasm [4] based on its subcellular localization. However, this localization-dependent regulatory network requires more experimental validation.

Furthermore, studies indicate that, besides BmNPV, the Helicoverpa armigera nucleopolyhedrovirus (HaNPV) Ha-VP39 can bind actin directly and polymerize it to drive retrograde nucleocapsid transport without cofactors [80], thereby reconfiguring the cytoskeleton for motility. Yet, it lacks intrinsic nuclear localization signals and depends on host factors for nuclear entry [81,82]. The precise molecular mechanism of the interaction between BmNPV VP39 and host F-actin remains unclear and warrants further investigation.

At the mitochondrial level, the host protein BmANT interacts with BmHSP60 to suppress viral replication [54], whereas the viral protein *LEF-11* hijacks the host ATAD3A/HSPD1 to activate energy metabolism. Determining if *LEF-11* directly interacts with BmANT represents a promising research avenue. Host Hsp90 promotes BmNPV proliferation by interacting with actin-like 4 (Actin-4) and enhancing viral gene expression [83], yet K64 acetylation inhibits replication [84]. E3 ubiquitin ligases also form complexes with HSP90 to ubiquitinate substrates.

Overall, the protein interaction mechanisms between BmNPV and *Bombyx mori* involve multiple pathways, but their synergistic or antagonistic relationships remain unclear. Future research should prioritize the following:

(i) Identifying critical viral hijacking nodes by utilizing multi-omics technologies to pinpoint key host molecular targets manipulated by the virus.
(ii) Developing targeted interventions by designing strategies against these targets, with safety and efficiency first validated in genetically modified silkworms or via genome editing.
(iii) Assessing the economic impact by evaluating the effects of any interventions on economically vital traits such as silk yield and quality to balance host health and agricultural value.

Although significant progress has been made in understanding the interactions between *Bombyx mori* and BmNPV, several key questions remain unresolved. Notably, the spatiotemporal dynamics of viral hijacking strategies—such as the dual functions of IAP1—and the host defense networks, particularly ncRNA-mediated metabolic reprogramming, are not yet fully defined. From an industrial perspective, translating these molecular insights into practical sericultural solutions faces several challenges, including balancing multi-gene editing for antiviral resistance with the maintenance of silk productivity and developing effective delivery systems for mitochondria-targeted antivirals. Addressing these challenges will require the integration of multi-omics profiling with high-throughput validation, leveraging this model system to enable scalable mechanistic exploration and agricultural application.

## Figures and Tables

**Table 1:** Comparison of protein interactions research methods.

Methodologies	Advantages	Limitations	Applicable Scenarios	Refs
Y2H	High throughput; can screen for unknown interacting proteins	High false positive rate; inability to detect membrane proteins or modified proteins	Large-scale screening of interactions; suitable for initial screening of intranuclear interactions	[31]
Co-IP	Close to physiological conditions; detectable natural complexes	Relies on specific antibodies; unsuitable for large-scale screening of interacting proteins	Validation of known interactions; applicable to physiological condition validation	[32]
GST pulldown	Controllable; suitable for in vitro validation	Non-physiological conditions; may bind non-specifically	In vitro validation of interactions; recombinant protein interaction studies	[33]
BiFC	Visualizing subcellular localization; can be used to study weak or transient interactions between proteins	Irreversible; fluorescence maturation may be delayed	Localization and validation of intracellular interactions	[34,35]
MS	High throughput, high specificity, and high flexibility	Equipment is expensive; complexity of data analysis	Structural analysis of protein complexes; localization of interaction interfaces	[36]
SPR	No marking is required; affinity and kinetic parameters can be determined	Equipment is expensive; protein needs to be purified	Quantitative analysis of the strength of interactions	[37]
AI-driven protein interaction prediction	Highly accurate prediction of protein structure	Limited support for dynamic interactions	Structure prediction and functional studies of protein complexes	[38]

**Table 2:** Apoptosis and cycle regulation.

Proliferative Effect on BmNPV	Interacting Proteins	Mechanisms	Refs.
Inhibits	ING5/P53	Promotes clearance of infected cells by accelerating apoptosis by reducing mitochondrial membrane potential	[39]
Promotes	Acetylated ING5/P53	Reduces P53 protein stability and inhibits its pro-apoptotic function	[41]
Promotes	BmIAP/BmPP5	Protein phosphorylation modification regulates apoptosis	[4]
Promotes	*LEF-11*/BmIMPI	Inhibition of CDK1/cyclin B activity leads to cell cycle arrest in G2/M phase	[42]
Promotes	IAP1/cyclin B	Inducing aberrant accumulation of cyclin B in the nucleus and specific blockade of the G2/M phase prolong the time window for viral assembly	[43]
Promotes	IAP1/BmCDK1	Reduces BmCDK1 levels, inhibits cell cycle progression, and supports viral replication and proliferation	[44]

**Table 3:** Regulation of viral protein invasion and transport.

Proliferative Effect on BmNPV	Interacting Proteins	Mechanisms	Refs
Promotes	GP64/SINAL10	K63-linked ubiquitination modification to stabilize GP64 conformation and enhance membrane fusion efficiency	[9]
Promotes	GP64/TRAP1	Enhanced membrane fusion efficiency	[46]
Promotes	GP64/BmREEPa-BmPtchd complex	Activates AMPK signaling via dephosphorylation, drives nuclear translocation, and upregulates viral proliferation-related genes	[47,48]
Mutant site Inhibits	GP64/NPC1-NPC2 receptor complex	Mediated viral endocytosis synergizes with membrane fusion and mutated reciprocal sites to inhibit proliferation	[50,51]
Promotes	SEC61/viral protein	Mediates translocation of viral proteins to the endoplasmic reticulum, supports viral replication, and promotes translation and assembly of viral proteins	[12]
Inhibits	GP64/FABP1	Antagonizes E3 ubiquitinase activity, inhibits GP64 membrane fusion, and inhibits viral membrane fusion and invasion	[12]
Inhibits	VP39/F-actin	Interaction with F-actin may interfere with viral transport (mechanism not defined)	[52]
Promotes	BmHsp90/BmTbce	Regulation of nucleocapsid–microtubule transport drives nuclear import for viral genome replication/transcription	[53]
Promotes	BmHsp90/BmGolga5	Interactions disrupt Golgi apparatus function, impacting viral protein processing/transport and viral particle assembly/release	[54]
Promotes	PK1/BmPP5/BmTFEB	Activates AMPK signaling via dephosphorylation, drives nuclear translocation, and upregulates viral proliferation-related genes	[55]
Promotes	BmE74A/viral protein	Directly binds to viral proteins, enhances viral gene expression, and promotes early viral gene transcription	[56]
Promotes	BmCdc37/BmHsp90	Enhances Hsp90 activity as a molecular chaperone, supports viral protein folding, and maintains viral protein function and stability	[57]
Promotes	PE38/BmeIF4E/BmSRPK	Interacts with translation factors and splicing kinases to promote early viral gene expression	[58]
Promotes	P74/JAB-MPN structural domain protein	Binding of midgut cell JAB-MPN protein mediates ODV invasion to promote virus infection and spread in the midgut	[59]
Inhibits	BmTHY/actin	Interferes with viral transport by binding actin; inhibits capsid migration and replication	[60]

**Table 4:** Non-coding RNA regulation.

Proliferative Effect on BmNPV	Interacting Proteins	Mechanisms	Refs
Promotes	lincRNA_XR209691.3/BmHSP70	Binding to the actin structural domain of BmHSP70 enhances its stability, optimizes viral protein folding efficiency, and promotes viral replication	[18]
Promotes	lnc557/BmELAVL1	Promotes viral mRNA (e.g., ie-1, gp64) stability and accelerates viral structural protein synthesis	[61]
Promotes	Lnc_209997/miR-275-5p	BmNPV infection downregulates Lnc_209997, releasing miR-275-5p to promote viral proliferation via signaling pathways	[62]
Inhibits	miR-3351/BmGSTe6 (A35)	Targets BmGSTe6 to regulate glutathione metabolism and inhibit viral proliferation	[63]
Inhibits	miR-6498-5p/BmPLPP2	miR-6498-5p inhibits viral replication by maintaining higher PLP levels through the inhibition of BmPLPP2	[64]

**Table 5:** Metabolic regulation.

Proliferative Effect on BmNPV	Interacting Proteins	Mechanisms	Refs
Inhibits	BmANT/BmHSP60	Interacts with BmHSP60 to downregulate its expression and inhibit viral replication; BmANT leads to abnormal accumulation of ANT and triggers ATP/ADP transport disorders	[54,67]
Inhibits	Bmcytc/Bmapaf/Bmcaspase-Nc	Release triggers the mitochondrial apoptotic pathway (Bmapaf/Bmcaspase-Nc) to clear infected cells	[68]
Promotes	p35/BmVDAC2-BmRACK1	Blocks cytochrome c release, inhibits mitochondria-dependent apoptosis, and promotes viral proliferation	[69]
Promotes	*LEF-11*/ATAD3A/hspd1 (hsp60)	Activation of ATPase activity and enhancement of mitochondrial OXPHOS support viral DNA replication	[70]

**Table 6:** Immunomodulation.

Proliferative Effect on BmNPV	Interacting Components	Mechanisms	Refs
Inhibit	BmTCTP/BmILF	Activates downstream immune signaling and significantly inhibits viral replication	[71]
Inhibit	BmSerpin3/storage protein	Involved in immunomodulation, regulating immune homeostasis, and indirectly suppressing viruses	[72]
Inhibit	BmSerpin3/SP7	Inhibition of SP7 modulates the PPO activation cascade and balances the intensity of the blackening reaction	[73]
-	serpin-1a/serpin-6/CLIP2	Serpin-1a and serpin-6 synergize to precisely regulate CLIP2 activity and balance immune activation	[77]

## Data Availability

Not applicable.
